# Assessment of a Mobile Health iPhone App for Semiautomated Self-management of Chronic Recurrent Medical Conditions Using an N-of-1 Trial Framework: Feasibility Pilot Study

**DOI:** 10.2196/34827

**Published:** 2022-04-12

**Authors:** Archana Mande, Susan L Moore, Farnoush Banaei-Kashani, Benjamin Echalier, Sheana Bull, Michael A Rosenberg

**Affiliations:** 1 Division of Personalized Medicine and Biomedical Informatics University of Colorado Anschutz Medical Campus Aurora, CO United States; 2 mHealth Impact Laboratory University of Colorado Anschutz Medical Campus Aurora, CO United States; 3 College of Engineering and Applied Science University of Colorado Denver Denver, CO United States; 4 Clinical Research Support Team University of Colorado Anschutz Medical Campus Aurora, CO United States

**Keywords:** mHealth, patient-specific modeling, chronic disease, smartphone, implementation and deployment, facilitators and barriers

## Abstract

**Background:**

Management of chronic recurrent medical conditions (CRMCs), such as migraine headaches, chronic pain, and anxiety/depression, remains a major challenge for modern providers. Our team has developed an edge-based, semiautomated mobile health (mHealth) technology called iMTracker that employs the N-of-1 trial approach to allow self-management of CRMCs.

**Objective:**

This study examines the patterns of adoption, identifies CRMCs that users selected for self-application, and explores barriers to use of the iMTracker app.

**Methods:**

This is a feasibility pilot study with internet-based recruitment that ran from May 15, 2019, to December 23, 2020. We recruited 180 patients to pilot test the iMTracker app for user-selected CRMCs for a 3-month period. Patients were administered surveys before and after the study.

**Results:**

We found reasonable usage rates: a total of 73/103 (70.9%) patients who were not lost to follow-up reported the full 3-month use of the app. Most users chose to use the iMTracker app to self-manage chronic pain (other than headaches; 80/212, 37.7%), followed by headaches in 36/212 (17.0%) and mental health (anxiety and depression) in 27/212 (12.8%). The recurrence rate of CRMCs was at least weekly in over 93% (169/180) of patients, with 36.1% (65/180) of CRMCs recurring multiple times in a day, 41.7% (75/180) daily, and 16.1% (29/180) weekly. We found that the main barriers to use were the design and technical function of the app, but that use of the app resulted in an improvement in confidence in the efficiency and safety/privacy of this approach.

**Conclusions:**

The iMTracker app provides a feasible platform for the N-of-1 trial approach to self-management of CRMCs, although internet-based recruitment provided limited follow-up, suggesting that in-person evaluation may be needed. The rate of CRMC recurrence was high enough to allow the N-of-1 trial assessment for most traits.

## Introduction

Chronic recurrent medical conditions (CRMCs) encompass a major proportion of the modern health care burden, accounting for significant costs in the form of both management and lost productive time [1]. For example, chronic migraine headaches affect about 2% of the global population [2], and in the United States alone, cost more than US $20 billion annually [1] to manage. Chronic low back pain accounts for over 5 hours/week in lost productivity by workers, resulting in over US $10 billion in lost revenue per year [1]. Mental health disorders, including depression and anxiety, accounted for 183.9 million disability-adjusted life years and 175.3 million years lived with disability worldwide [3], with an increase of 37.6% over the years from 1990 to 2010 [3].

On a more granular level, CRMCs create a major challenge for today’s busy clinician. Although widely variable across providers and practices, the time available for a face-to-face encounter with patients continues to trend downward, despite an increase in the number of clinical items needing to be addressed [4]. As a result, providers have less time available to focus on the range of triggers and contributing factors for any given CRMC. This trend is unfortunate, as for many CRMCs the number and complexity of environmental and lifestyle triggers can be quite robust. For example, sleep changes have been described in about 50% of patients with migraine headaches, although 75% of patients also chose to sleep due to the migraine headache [5]. In addition, a study of 1207 patients with migraine headache identified no less than 16 possible triggers present in at least 5% of these individuals [6]. A similar scale in triggers has also been noted for depression [7], anxiety [8], and chronic low back pain flares [9]. As such, tailored management of patients with these and other CRMCs often requires the provider to take a detailed, longitudinal history with attention to temporal relationships—an approach that fits poorly with the practical constraints of modern clinical practice.

**Figure 1:**
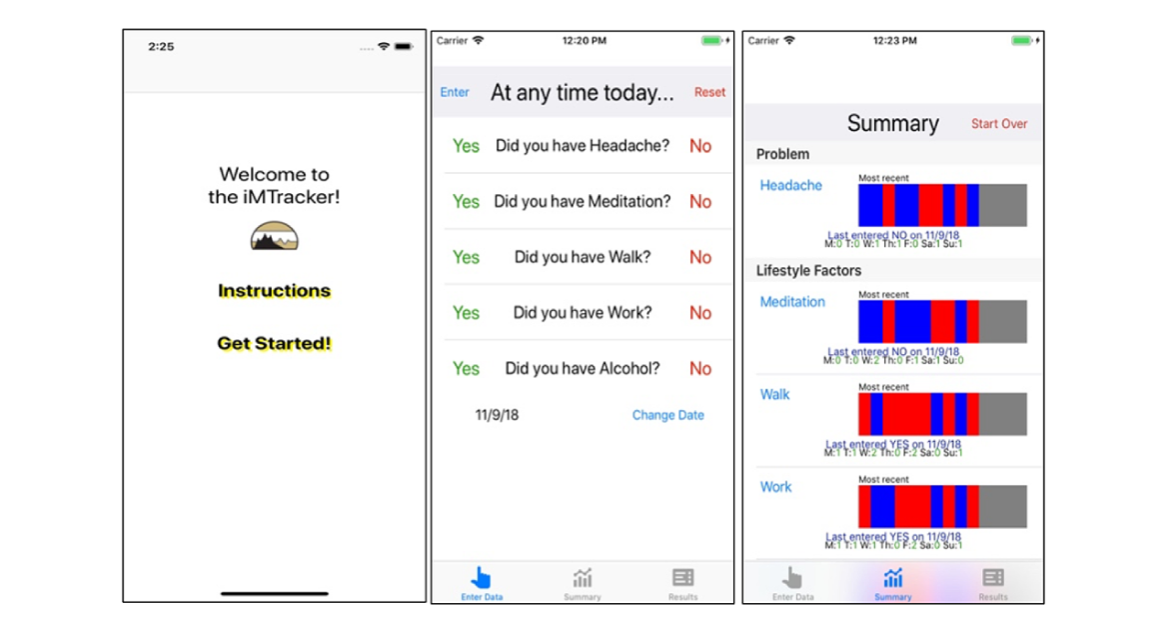
Screenshots of iMTracker.

Despite these limitations, there is evidence that an individualized approach to self-management of CRMCs using mobile health (mHealth) apps has potential to improve clinical outcomes. Specifically, the N-of-1 approach to care has been applied to study various interventions for pain [10-14], depression [15-17], anxiety [18,19], and migraine headaches [20-22], and has been incorporated into mHealth technology [23-25]. In 2017, our team developed a prototype semiautomated iOS mHealth app called the iMTracker (Figure 1), which incorporates the N-of-1 platform for patient-entered data to log recurrences of a given CRMC, as well as the opportunity to log possible triggers or suppressors of the CRMC. The iMTracker provides edge-based analysis of symptom correlations, in which data are stored and analyzed on each user’s device, without the need to transfer or store data to a central server. However, it is unknown which specific patients with CRMCs would be most likely to use the iMTracker for self-management, and whether the rate of recurrence is high enough to maintain a sufficient level of engagement to draw meaningful associations with lifestyle triggers and to evaluate the impact of interventions on recurrence rate. In this feasibility pilot investigation, we aimed to apply an internet-based recruitment approach to enroll patients to trial the iMTracker app. Our goal in this study was to examine the 3-month adoption rate of the iMTracker app by patients with CRMCs, to understand the patterns and characteristics of the possible CRMCs and users, and to identify design and functional barriers to the use of iMTracker prior to its use. Additionally, we planned to examine the strengths and limitations of the internet-based recruitment approach to development and testing of mHealth apps, and identify areas to address for future prospective studies aimed at improving outcomes.

## Methods

### Patients

From May 15, 2019, to December 23, 2020, we recruited 180 patients to test the iMTracker app for iPhone for self-management of their CRMCs using an internet-based study design. Inclusion criteria were aged 18 or older, presence of a CRMC, and use of an iPhone. There were no official exclusion criteria, although based on study design and app functionality, patients generally needed to be English speaking and familiar with the use of iPhone apps, as well as the use of email and internet. We started with advertising on social media, such as Twitter, campus-based fliers, and provider word-of-mouth, but found limited recruitment, by which only 2 patients were recruited. We then employed the TrialFacts patient-recruitment company [26] (San Diego, CA, USA) to assist with internet-based recruitment. Patients were provided a small financial stipend for participation, which was paid on enrollment only (nothing additional for follow-up). Written informed consent was obtained for all patients.

### Ethics Approval

The study protocol was approved by the University of Colorado Institutional Review Board (Protocol #18-1000).

### mHealth App (iMTracker)

**Figure 2:**
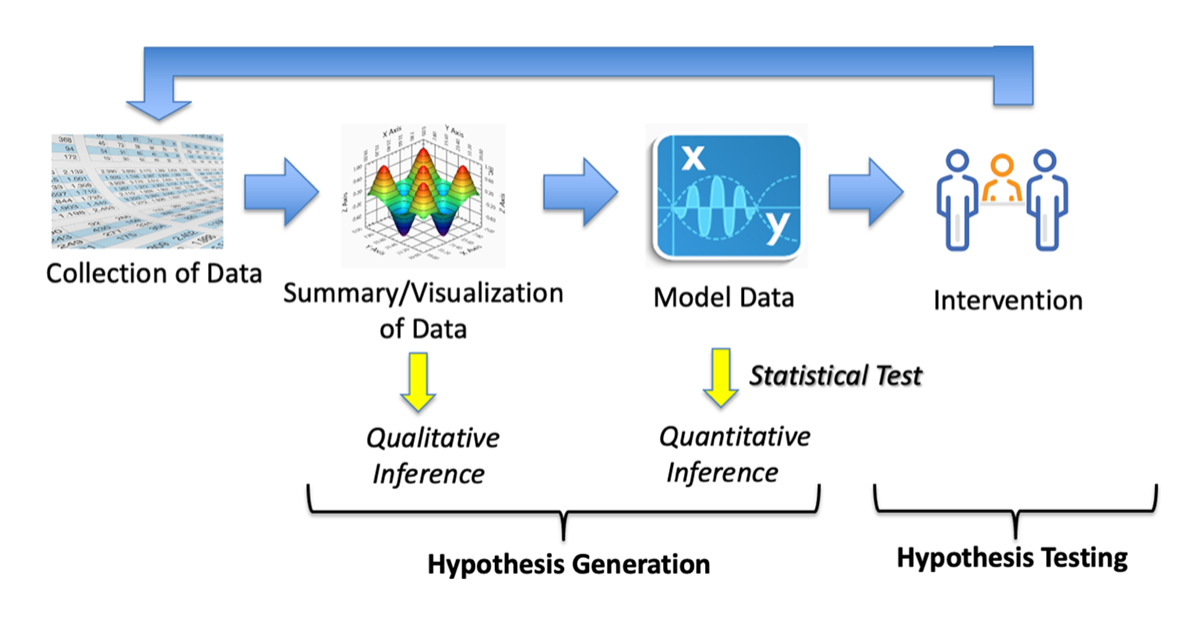
The semiautomated N-of-1 approach motivating the iMTracker design.

The platform of iMTracker was designed based on an automated N-of-1 trial approach (Figure 2) that includes both hypothesis generation and hypothesis testing, which can be built into the logic of an mHealth app. The iMTracker allows the user to select any problem (outcome) and any potential lifestyle factors (risk factors) or intervention that the user would like to test for an association with the outcome. Through iteration between hypothesis generation (ie, “Is there an association between risk factor A and occurrence of my condition?”) and hypothesis testing (ie, “Does changing risk factor A improve the rate of occurrence of my condition?”), the user is able to self-manage his or her condition toward an overall goal of reducing recurrence. The platform thus provides a semiautomated approach to self-management, in which the analysis provides potential lifestyle/behavioral factors that are associated with the CRMC, but allows users to select which factor to intervene upon to examine impact on CRMC recurrence rate. Importantly, the overarching design of the iMTracker app has been focused on application of edge computing [27,28] strategies that run on the mobile device itself, to allow complete usage of the iMTracker app without the need for transfer or storage of data on a server, which provides patients with a level of privacy and data security [29,30].

Data are manually entered by the user, presented in a visual format, and then modeled for correlations between the selected outcome (problem) and potential risk factors (Figure 1). Through built-in notifications, the iMTracker app prompts the user to input data daily, and keeps a running summary of the inputs. This analysis includes correlation between the outcome and risk factors on a daily basis and with 1-day lag to identify risk factors that could potentially cause the outcome on the following day, using the phi statistic for correlation between discrete variables [31]. Although analysis is performed after only 3 days of data collection, users are informed that the accuracy of the correlation is higher with greater amounts of data collection (Multimedia Appendix 1). Once enough data have been collected to form hypotheses about causative associations, users are directed to reset the data collection and select an intervention in the form of a lifestyle modification, from which future data will examine the role of that intervention in reducing recurrences of the outcome.

### Survey

Our team designed a brief survey instrument with several goals in mind. First, we wanted to identify which specific CRMCs users selected for self-management using the iMTracker app. These diagnoses were self-provided, and we did not perform a separate validation with either treating clinicians or chart review. Second, we sought to collect information about the typical pattern of CRMCs—that is, frequency of recurrence—to understand the burden of disease of a possible user of the iMTracker app, and also to guide future work in automating analyses toward sufficient statistical power to detect associated lifestyle conditions and the effect of interventions. Third, we included questions aimed at detecting prior experience with regular data collection (eg, “How often do you weigh yourself?”), information sharing (eg, “How often do you post on social media?”), and electronic engagement with providers using email or secure messaging. Broadly, these questions helped to frame the users’ motivation for using this type of technology for self-management of CRMCs. Fourth, we inquired about concerns of using technology for self-management of conditions; specifically, we asked users to rank concerns related to data security, privacy, efficiency (time demand), and efficacy. Finally, we inquired about specific concerns with the iMTracker app, and asked for qualitative input about the design and function. In addition, we collected basic demographic information about categories of age, education, race, and ethnicity.

The follow-up survey was designed to obtain information about the app itself, as well as the process of the N-of-1 approach to self-management of their selected condition, and to assess the 3-month adoption rate to provide a baseline for future clinical trials. Patients were sent a link to the poststudy survey 3 months after the date of enrollment. Patients that provided no answer for the 3-month adoption, but who completed the postsurvey were assumed to have not completed the 3-month adoption. The postsurvey questions are provided in Multimedia Appendix 2.

The study was conducted remotely using email and phone calls with patients. After informed consent was obtained, patients were given a link to the online survey using a REDCap database. Patients were guided through download and use of the iMTracker mHealth app for iPhone (iOS) by a member (AM) of the research team, and given the opportunity to provide qualitative feedback about the app design, outside the survey data. The postuse survey was deployed after 3 months of use (Multimedia Appendix 3).

### Analysis

All study data were collected in a REDCap database. Statistical tests of proportions were based on Fisher exact test. The analysis was performed using Stata IC, version 16.1 (StataCorp, Inc.).

## Results

Of the 180 patients who completed the preuse survey, 103 also completed the postuse survey (57.2%). Only 2 patients were recruited by the study team outside of use of the TrialFacts company referrals. A total of 172 patients (95.6%) were under the age of 65, with the predominant age range being 31-45 (80 patients, 44.4%; Table 1). Most patients had at least some college (171/180, 95.0%), and most were White (144/180, 80.0%) and non-Hispanic/Latino (161/180, 89.4%).

The most common CRMCs (self-reported) for which patients planned to use the iMTracker app to self-manage were pain (80/212, 37.7%), including low back pain and other musculoskeletal pain syndromes; headaches (36/212, 17.0%), including migraines; gastrointestinal symptoms (17/212, 8.0%), including inflammatory bowel disease flares and irritable bowel disease; and mental health conditions, including anxiety (12/212, 5.7%) and depression (15/212, 7.1%; Table 2). A total of 19/180 patients (10.6%) planned to use the app to monitor more than 1 CRMC. For most CRMCs, frequency was daily (75/180, 41.7%) or multiple times a day (65/180, 36.1%), with few occurring less often than monthly (5/180, 2.8%; Table 3). Patients were allowed to apply the iMTracker app to more than 1 condition, which is why there are 212 listed in Table 2.

To assess overall patterns of self-management and use of media, patients were asked about lifestyle and technology savviness. About one-sixth (28/180, 15.6%) weighed themselves daily, 58/180 (32.2%) weighed themselves at least weekly, 42/180 (23.3%) weighed themselves monthly, and 52/180 (28.9%) weighed themselves rarely or not at all. A total of 49/180 (27.2%) posted on social media multiple times a day, 54/180 (30.0%) posted daily, 49/180 (27.2%) posted weekly, 12/180 (6.7%) posted monthly, 14/180 (7.8%) posted rarely or never, and 2/180 (1.1%) were not on social media; 64/178 (36.0%) communicated with their primary physician regularly using messaging/technology, 66/178 (37.1%) communicated rarely, and 13/178 (7.3%) preferred not to communicate using technology/messaging and only in person. Prior to the study, 74/180 patients (41.1%) said they were very likely to use an mHealth app to self-manage CRMCs, 63/180 (35.0%) were somewhat likely, and 6/180 (3.3%) said they were unlikely to use an mHealth app to self-manage CRMCs. After using the iMTracker app, all patients who said they were unlikely to use an mHealth app changed their answers to neutral (2/6, 33%) or somewhat likely (4/6, 67%).

Patients were asked about concerns for using an mHealth app for self-management of CRMCs both before and after use of the iMTracker. As shown in Table 4, patients were generally more likely to have concerns about effectiveness after using the app, and less likely to have concerns about privacy, data safety/security, or time requirements, after use.

Of the 103 patients who completed the postuse survey, 73 (70.9%; Table 5) said they used the iMTracker app for the planned 3-month period; among those who stopped beforehand, 2/16 (13%) used it for 2 months, 5/16 (31%) used it for 1 month, and 9/16 (56%) used it for less than a month. Among those completing the 3-month use period, only 3/103 (2.9%) failed to enter data on over 50% of days, and 22/103 (21.4%) reported missing less than 5% of days entering data. These patients reported reviewing their data summary weekly or every few weeks in 13/22 (59%) cases, and daily in 3/22 (14%) cases. Finally, 27/103 (26.2%) patients said they were likely or very likely to use the iMTracker app again to self-manage their CRMCs, and 58/103 (56.3%) said they were unlikely to use it without modifications. There was a potential signal for increased levels of education being statistically associated with increased 3-month adoption rate (*P*=.04; Table 5), although the association did not reach a level of statistical significance after adjustment for multiple comparisons (Bonferroni *P* for significance [α]=0.05/4=.0125). Among the subjective reasons for not continuing to use the iMTracker app, issues with data sharing and ease of use were most cited, followed by design/display limitations.

**Table 1:** Demographics of iMTracker users.

Demographics		Value, n (%)
**Age (years; n=180)**		
	Under 30	38 (21.1)
	31-45	80 (44.4)
	46-55	32 (17.8)
	56-65	22 (12.2)
	66-75	8 (4.4)
	Over 75	0 (0)
**Education (n=177)**		
	Grade school only	1 (0.6)
	High-school diploma/general educational development	5 (2.8)
	Some college	50 (28.2)
	College degree	66 (37.3)
	Master’s degree	48 (27.1)
	Doctorate degree	7 (3.9)
**Race (n=180)**		
	White	144 (80.0)
	African American	14 (7.8)
	Asian	9 (5.0)
	American Indian or Alaskan Native	5 (2.8)
	More than 1/unknown	8 (4.4)
**Ethnicity (n=180)**		
	Hispanic/Latino	17 (9.4)
	Not Hispanic/Latino	161 (89.4)
	Unknown	2 (1.1)

**Table 2:** Groups of chronic recurrent medical conditions for which patients planned to use the iMTracker app (n=212).

Condition group	Frequency, n (%)
Chronic pain	80 (37.7)
Headaches	36 (17.0)
Gastrointestinal symptoms	17 (8.0)
Depression	15 (7.1)
Anxiety	12 (5.7)
Palpitations	7 (3.3)
Hypertension	7 (3.3)
Dizziness	5 (2.4)
Other	33 (15.6)

**Table 3:** Frequency of recurrence (n=180).

Recurrence rate	Frequency, n (%)
Multiple times/day	65 (36.1)
Daily	75 (41.7)
Weekly	29 (16.1)
Monthly	6 (3.3)
Every few months	3 (1.7)
Less than every few months	2 (1.1)

**Table 4:** Change in concerns about mHealth apps for self-management of chronic recurrent medical conditions (n=103).

Concern	More likely, n (%)	Less likely, n (%)	Significance (*P* value)
Effectiveness	28 (27.2)	10 (9.7)	.001
Privacy	8 (7.8)	11 (10.7)	<.001
Data safety/security	5 (4.9)	18 (17.5)	.001
Time demands	11 (10.7)	29 (28.2)	.005

**Table 5:** Poststudy survey.

Characteristics^a^		Completed follow-up, n/N (%)	3-month adoption, n/N (%)^b^
**Age category (n=103/180)**			
	Under 30	19/38 (50)	13/19 (68)
	31-45	46/80 (58)	29/46 (63)
	46-55	20/32 (63)	15/20 (75)
	56-65	13/22 (59)	11/13 (85)
	66-75	5/8 (63)	5/7 (71)
	*P* value	.87	.37
**Education (n=101/180)**			
	Grade school only	0/4 (0)	N/A^c^
	High school diploma/general educational development	1/5 (20)	1/1 (100)
	Some college	22/50 (44)	17/22 (77)
	College degree	39/66 (59)	28/39 (72)
	Master’s degree	34/48 (71)	22/34 (65)
	Doctorate degree	5/7 (71)	3/5 (60)
	*P* value	.04	.83
**Race (n=103/180)**			
	Caucasian	84/144 (58)	60/84 (71)
	African American	9/14 (64)	8/9 (89)
	Asian	2/9 (22)	2/2 (100)
	American Indian or Alaskan Native	3/5 (60)	3/3 (100)
	More than 1/unknown	5/8 (63)	3/5 (60)
	*P* value	.37	.05
**Ethnicity (n=103/180)**		N=103	
	Hispanic/Latino	10/17 (59)	7/10 (70)
	Not Hispanic/Latino	92/161 (57)	65/92 (71)
	Unknown	1/2 (50)	1/1 (100)
	*P* value	>.99	>.99

^a^N=103 for age category, race, and ethnicity, and 101 for education.

^b^N=73 for age category and ethnicity, 71 for education, and 76 for race.

^c^N/A: not applicable.

## Discussion

### Principal Findings

In this internet-based, pilot study of predominantly young and middle-age, educated, White patients, using a semiautomated, edge-based, mHealth app that uses individualized data for tailored management of CRMCs, we made several key observations with regard to both the internet-based recruitment approach to the study of mHealth apps, as well as the specific usage rates and patterns of use by participants. First, we found that while an internet-based recruitment approach was superior to “grass-roots” local methods of recruiting participants on our campus using fliers and word-of-mouth, the 3-month follow-up rates were only slightly above 50% (103/180), indicating that future studies using this type of methodology targeted to achieve a prespecified degree of statistical power will need to account for a high number of dropouts. Second, we found that among those with complete follow-up, the 3-month adoption rate of the iMTracker app was about 70.9% (73/103), with the most common CRMCs that users chose to self-apply the app being chronic pain, headaches, and mental health conditions. This information not only provides conditions and clinical settings in which to target future clinical trials, but also indicates that there may be a need for better tools to manage these conditions beyond what is presently available in clinical practice. Importantly, we found that on completion of this study, more patients had increased perceptions of the safety, privacy, and time demands with the use of an mHealth app for self-management of CRMCs. Finally, we found that the main barrier to use, based on both subjective and quantitative feedback, was related to the design and workflow of iMTracker itself, which was reflected in the decrease in perception of efficacy noted on completion, and has important implications for future development and testing of this app as well as other mHealth technologies. In other words, this finding indicates that patients are likely to be receptive to the semiautomated N-of-1 trial methodology employed by the iMTracker app, but that greater attention to design and function is needed before moving forward with clinical testing targeted toward improvement in outcomes.

mHealth apps have increased significantly in frequency over the years, with iOS apps including health and fitness groups increasing from 43,000 in 2013 to 98,000 in 2015 [32]. Unfortunately, these tools do not consistently employ best practices for self-management [33], and many of these approaches have failed to reach any meaningful level of adoption across the medical community [32,34], likely due to a lack of formal clinical testing. Our finding that there was an increased concern among patients about the effectiveness of iMTracker is consistent with prior studies of mHealth app for self-management of CRMCs. In a previous investigation, it was found that only 3.4% of apps on the iTunes and Google Play stores promoted for management of depression and anxiety had research to justify their claims of effectiveness, with only 30.4% having expert input in development [35]. A study by Devan et al [36] of 19 apps available commercially for self-management of pain found that only 2 had been validated to improve health outcomes. A similar lack of scientific support for commercially available mental health–targeted [37-39] and pain [25,40] apps has been reported by other investigators. Although we did not inquire about prior use of mHealth apps for self-management, one can infer that most participants in this study had tried prior apps without success. Clinical validation of any mHealth app should be required before integration into the clinical care process, and our study further suggests that while users are optimistic that self-management using an app is possible, follow-up clinical studies will be needed.

Among the characteristics of the specific CRMCs that patients identified for use of an mHealth app, recurrent pain, headaches, and mental health were highly represented. While these diagnoses were self-identified by users, and not validated with clinicians or clinical data (ie, chart review), it does help to identify potential clinics and providers for testing mHealth apps, as would be needed before an app such as the iMTracker could be incorporated into routine clinical care. In addition, the majority of patients noted a high frequency of recurrence of their condition, which is key in determining the number of patients needed for a prospective study to demonstrate efficacy, as a condition that occurred less frequently, for example, once or twice a year, would require an extended amount of time to identify correlation of episodes with other lifestyle and behaviors, or assess the impact of an intervention.

Although our study did not specifically examine clinical efficacy of the iMTracker app in terms of a reduction in the primary CRMC, we did note that there was enthusiasm about future uses, particularly if barriers related to design and function could be addressed. This finding is in line with prior work, such as that by Neuhauser et al [41,42], who noted that participatory methods linked with traditional health communication theory and methods can create effective health communication using artificial intelligence, highlighting the role of design science theory in the development and refinement of mHealth apps. Such insights highlight the challenge that is unique to mHealth, and other health IT apps, in which consideration of user-based preferences and desires must be merged with information and guidance grounded in biology and evidence-based medicine principles. In terms of design life cycles, this requires an integrated design approach with features of both top-down (ie, waterfall) strategies and bottom-up, user-driven design (eg, agile) strategies. Our team has already begun efforts to improve the design and function of the iMTracker app, and future studies will examine the improvement in these changes to make the app more in line with the level of commercial design that many patients have come to expect from all mobile apps, in addition to mHealth.

Despite results to suggest a high degree of potential for clinical application of the iMTracker app, there were several key limitations in our study. First, we generally had a limited amount of follow-up of patients, with roughly half of the patients who enrolled being lost to follow-up. We suspect that this limitation highlighted the challenges of using the internet for recruitment, and the trade-off between use of network-based recruitment methods (ie, online) and in-person clinic recruitment. As an extreme example of the potential of the former, the Apple Watch study recruited over 400,000 participants in an 8-month period using online methods, although only 450 actually returned the confirmatory patches in follow-up [43]. As such, future studies of mHealth technology should consider that the potential benefit in terms of recruitment numbers using internet-based recruitment may accompany a relatively high degree of dropouts. A second limitation was that the population we studied were primarily White, educated, and young/middle age adults; these were individuals who engage regularly with providers using technology, post to social media, and perform self-management with regular weight checks. Missing from our population are older patients, those with less education, and those from underrepresented populations—the type of populations that have also been shown to have less close clinical follow-up for their conditions [44-46], and who might stand to benefit the most from an app that allows self-management. This population bias is critical in considerations of further app development as the design and functionality changes that would typically guide app development would be needed for successful integration with clinical care. Further work is needed on methods to include less represented populations in mHealth studies. Among the issues with biased recruitment was the omission of sex from the baseline variables we collected. Qualitatively (based on the first names of the participants), we suspect recruitment was not heavily imbalanced toward 1 specific sex, although formal assessment would have been beneficial in terms of statistical analysis. In future studies, we plan to examine sex along with other user characteristics in terms of both usage patterns and study participation. Finally, a key limitation of the study was the inability to confirm diagnoses or response to therapy among users of iMTracker. While we have ongoing studies to examine the app prospectively toward clinical outcomes, the findings from this investigation provided important information about which specific conditions, and which types of clinics, to target for recruitment. This finding was critical as the iMTracker was originally developed toward treatment of recurrent cardiac symptoms (ie, palpitations), and yet we identified other conditions that included chronic pain, headaches, and mental health as being more heavily favored by patients in this investigation.

In future studies we plan to examine the impact of specific design and function improvements on iMTracker, including an examination of the use of industry-standard Agile development life cycles to make iterative improvements to the user interface within the Scrum methodology [47]. Our team has already employed a newer user interface and data entry design based on emojis, although additional work is needed to ensure all users of all levels of education and medical literacy can use the app comfortably. Following this step, we plan to target clinical trials of iMTracker to primary care, neurology, and psychiatry clinics to assess the impact of N-of-1 management on recurrence of chronic pain, headaches, and mental health conditions. Finally, our team has continued work on integration of more sophisticated data management approaches using federated and distributive learning, as well as Bayesian-based analytical frameworks, and we plan to examine the improvement in accuracy with these innovations. Ultimately, much work is needed before the iMTracker app can be used routinely in clinics to manage CRMCs, although feedback from this study has helped target efforts toward high-yield conditions and modifications to improve the chances of success.

### Conclusions

In conclusion, in this feasibility pilot study using internet-based recruitment, we found that the primary barrier to investigation was study follow-up, but that among those who were not lost to follow-up, there was generally good adherence to use of the iMTracker app. We identified design and function barriers as being of foremost concern among users, but also noted that the frequency of recurrence of the selected CRMCs should provide ample opportunity to identify a clinical benefit for future studies. We also identified population bias in the patients enrolled using internet-based recruitment alone, and note that additional efforts will be needed to ensure that future studies enroll sufficient numbers of underrepresented populations, specifically older, non-White, and less education populations.

## Appendix

Multimedia Appendix 1 Screenshot from Results presentation of iMTracker.

Multimedia Appendix 2 Postuse survey questions.

Multimedia Appendix 3 Sample of feedback about iMTracker from users on postsurvey.

## Abbreviations

CRMC: chronic recurrent medical condition
mHealth: mobile health
