# Supplementary figures and images for: Assessment of a Mobile Health iPhone App for Semiautomated Self-management of Chronic Recurrent Medical Conditions Using an N-of-1 Trial Framework: Feasibility Pilot Study
Source: JMIR Form Res. 2022 Apr 12;6(4):e34827. doi: 10.2196/34827 (PMC9044158; doi:10.2196/34827)

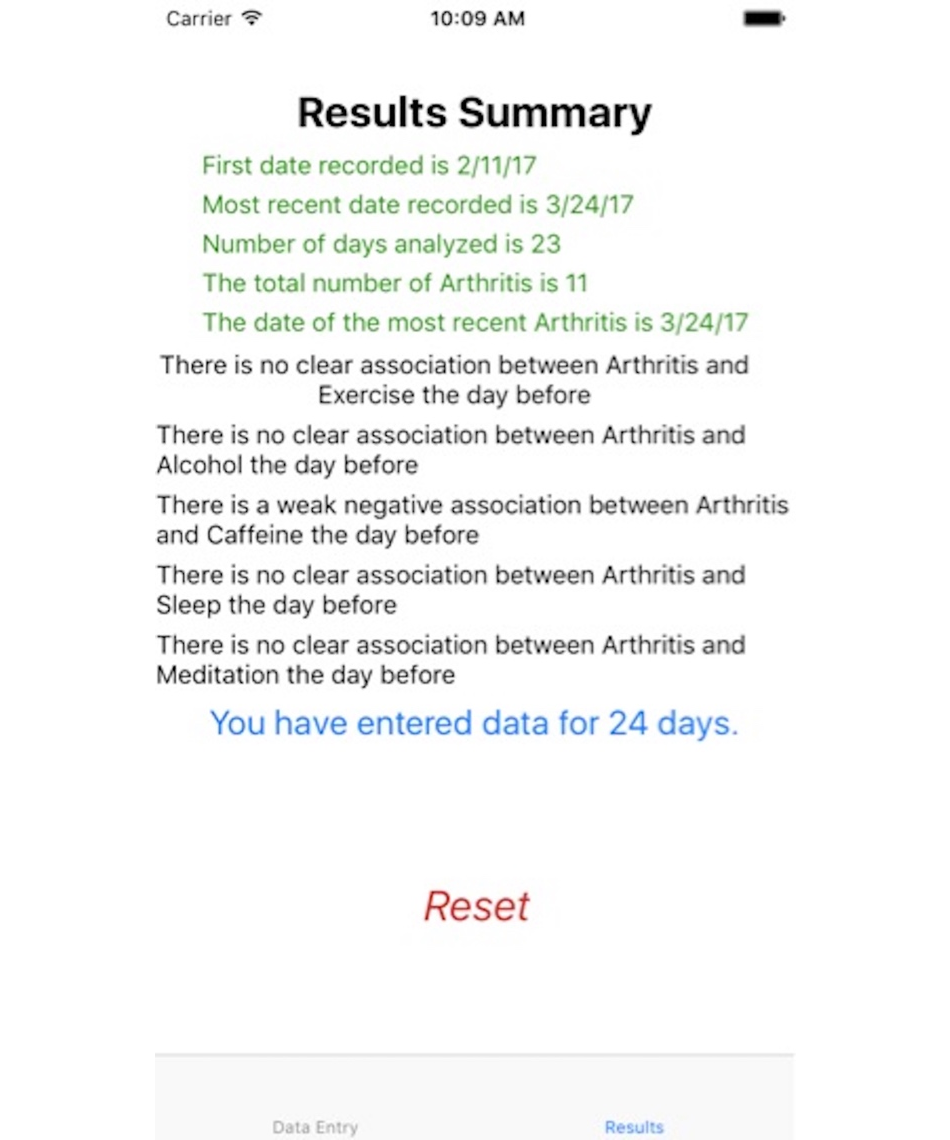

Supplement: Multimedia Appendix 1 [file formative_v6i4e34827_app1.png]
