# Supplementary material for: Assessment of a Mobile Health iPhone App for Semiautomated Self-management of Chronic Recurrent Medical Conditions Using an N-of-1 Trial Framework: Feasibility Pilot Study
Source: JMIR Form Res. 2022 Apr 12;6(4):e34827. doi: 10.2196/34827 (PMC9044158; doi:10.2196/34827)
Supplement: Multimedia Appendix 2 [file formative_v6i4e34827_app2.docx]

**Multimedia Appendix 2. Postuse survey questions.**

| Did you use the iMTracker to monitor your condition for at least 3 months? |
| --- |
| If no, then how long did you use the iMTracker to monitor your condition? |
| Which function did you use the iMTracker for? |
| How often did you forget to enter data for a day? |
| How often did you review your summary and/or results? |
| On a scale from 1-5 (1= no burden, 5= major burden), how much of a burden was entering data in the app? |
| On a scale from 1-5 (1= not helpful, 5= very helpful), how helpful was the analysis in terms of helping you understand the pattern of your condition? |
| On a scale of 1-5 (1= not helpful, 5= very helpful), how helpful was the analysis in terms of helping you identify associations with your condition? |
| On a scale of 1-5 (1= not helpful, 5= very helpful), how helpful was the analysis in terms of helping you to make lifestyle changes to reduce the burden of your condition? |
| How often did you experience your symptom during the study period? |
| How often did you share your data for the study with the study team? |
| How often did you review your summary and/or results? |
| On a scale of 1-5 (1= no burden, 5= major burden), how much of a burden was entering data in the app? |
| On a scale of 1-5 (1= not helpful, 5= very helpful), how helpful was the analysis in terms of helping you understand the pattern of your condition? |
| On a scale of 1-5 (1= not helpful, 5= very helpful), how helpful was the analysis in terms of helping you identify associations with your condition? |
| On a scale of 1-5 (1= unlikely, 5= very likely), how likely would you be to use the iMTracker after this study to manage this condition? |
| On a scale from 1-5 (1 = unlikely, 5 = very likely), how likely would you be to use the iMTracker after this study to manage other conditions? |
| Which condition would you use it for? |
| If you could change anything about the iMTracker, what would it be? |
| Do you have any concerns about the iMTracker being used for health care? |
