# Supplementary material for: Assessment of a Mobile Health iPhone App for Semiautomated Self-management of Chronic Recurrent Medical Conditions Using an N-of-1 Trial Framework: Feasibility Pilot Study
Source: JMIR Form Res. 2022 Apr 12;6(4):e34827. doi: 10.2196/34827 (PMC9044158; doi:10.2196/34827)
Supplement: Multimedia Appendix 3 [file formative_v6i4e34827_app3.docx]

**Multimedia Appendix 3. Sample of feedback about iMTracker from users on postsurvey**.

| **If you could change anything about the iMTracker, what would it be?** |
| --- |
| Honestly, the interface is not at all user-friendly. I think making more visually pleasing graphics and a more complicated system would benefit it. |
| more effective ways to enter data |
| I think the UI needs to be changed. That was the biggest issue, buttons were in weird places, how the app processed data was weird, and overall there were minor issues throughout the app that made it hard to use. I would use the app again, but with these issues it makes it hard to use it at times. |
| Streamline it. It is a good start. |
| The wording, visual (words overlap). Add a "notes" section for each daily page. |
| Would be helpful to visualize a calendar of when you entered and when it was forgotten |
| The graph charts and tracking aren't clear in understanding the results |
| Having to change the date. That is the clunky bit. Sometimes I would forget and overwrite the previous days data |
| More of a doctor connection to adjust what to track and what information used. |
| I wish it would auto populate the calendar or have the calendar selection be the screen before entering the data. It was frustrating to make my days selection for yes/no and have to reset it to make the calendar selection and then reselect the answers. |
| entering the data was confusing at first and didn't know i was doing it wrong for a long time |
| Nothing. App is fine but after I had consistent analysis info I lost interest in continuing to enter data. |
| It was hard to enter data...I kept accidentally deleting it. |
| Complete re-design. Not user friendly, not useable. |
| Being able to edit values to track along the way |
| Track multiple symptoms |
| i would make it a little more aesthetically appealing; the interface leaves a lot to be desired and things overlap that shouldn't. |
| Nothing. Easy to use and no issues to report. |
| The UI and UX were terrible. It hinders input by always verifying answers before going on to the next question, there's no account or home option to find any app or user information(ie-version number for this), there aren't back buttons for date entering and other screens. Overall, it was not user friendly and was a bigger hassle to input than a piece of paper would have been. |
